# CYP4B1 is a prognostic biomarker and potential therapeutic target in lung adenocarcinoma

**DOI:** 10.1371/journal.pone.0247020

**Published:** 2021-02-16

**Authors:** Xiaoling Liu, Yichen Jia, Changyuan Shi, Dechen Kong, Yuanming Wu, Tiantian Zhang, Anjie Wei, Dan Wang

**Affiliations:** 1 College of Basic Medicine, Jining Medical University, Jining City, China; 2 Institute of Medical Technology, Qiqihar Medical University, Qiqihar City, China; 3 Institute of Forensic Medicine and Laboratory Medicine, Jining Medical University, Jining City, China; Sapporo Ika Daigaku, JAPAN

## Abstract

CYP4B1 belongs to the mammalian CYP4 enzyme family and is predominantly expressed in the lungs of humans. It is responsible for the oxidative metabolism of a wide range of endogenous compounds and xenobiotics. In this study, using data from The Cancer Genome Atlas (TCGA) project and the Gene Expression Omnibus (GEO) database, a secondary analysis was performed to explore the expression profile of *CYP4B1*, as well as its prognostic value in patients with lung adenocarcinoma (LUAD). Based on the obtained results, a significantly decreased *CYP4B1* expression was discovered in patients with LUAD when compared with their normal counterparts (*p*<0.05), and was linked to age younger than 65 years (*p* = 0.0041), history of pharmaceutical (*p* = 0.0127) and radiation (*p* = 0.0340) therapy, mutations in *KRAS/EGFR/ALK* (*p* = 0.0239), and living status of dead (*p* = 0.0026). Survival analysis indicated that the low *CYP4B1* expression was an independent prognostic indicator of shorter survival in terms of overall survival (OS) and recurrence-free survival (RFS) in patients with LUAD. The copy number alterations (CNAs) and sites of cg23440155 and cg23414387 hypermethylation might contribute to the decreased *CYP4B1* expression. Gene set enrichment analysis (GSEA) suggested that *CYP4B1* might act as an oncogene in LUAD by preventing biological metabolism pathways of exogenous and endogenous compounds and enhancing DNA replication and cell cycle activities. In conclusion, *CYP4B1* expression may serve as a valuable independent prognostic biomarker and a potential therapeutic target in patients with LUAD.

## 1. Introduction

Non-small cell lung cancer (NSCLC) is one of the most frequently diagnosed tumors and the leading cause of cancer-related deaths worldwide, accounting for approximately 85% of lung cancer cases [1]. Lung adenocarcinoma (LUAD) and squamous cell carcinoma (LUSC) are the main histological subtypes of NSCLC, with the former representing more than 40% of lung cancers, gradually replacing the latter as the most frequent histological subtype in the past 25 years for unknown causes [2]. It is well known that cigarette smoking is by far the most important risk factor for lung cancer; however, LUAD is the most common type of lung cancer observed in non-smokers, indicating that multiple nonsmoking factors, including air pollution, radiation, and environmental related carcinogens, might also be responsible for LUAD carcinogenesis [3, 4].

Owing to the insignificant early symptoms and the tendency toward hematogenous metastasis, most patients with LUAD are diagnosed in the middle and late stages when diagnosed, thus missing the ideal period for diagnosis and treatment [5]. Combined with the poor sensitivity to radiochemotherapy, the mortality of patients with LUAD at 5 years is markedly high, ranging from 43% to 95%, depending on the stage [6]. In the last three decades, extensive efforts have been made in the early detection and treatment of LUAD to improve the patient situation, especially the application of genome-guided molecularly targeted therapy [*e*.*g*., anti-vascular endothelial growth factor (VEGF) bevacizumab and anti-epidermal growth factor receptor (EGFR) necitumumab therapy] and immunotherapy [*e*.*g*., pembrolizumab, an anti-PD-1 (programmed death-1) antibody]. However, to date, no comprehensive improvement in the 5-year survival of LUAD has been achieved. Therefore, the search for new and effective biomarkers to screen out high-risk patients and predict the prognosis of LUAD has become an essential part of lung cancer prevention and treatment.

Altered cellular metabolism is a prominent feature of tumorigenesis. Cytochrome P450 (CYP), a superfamily of cysteinato-heme monooxygenases, is an essential element of in this system. It serves as the most important phase I drug metabolism enzyme system, undertaking oxidative reactions in the body [7]. Furthermore, it is widely involved in the metabolic processes of exogenous drugs, environmental poisons, carcinogens, and endogenous hormones, thus playing a pivotal role in regulating the interaction between the organism and the external environment, as well as in maintaining the homeostasis *in vivo*. Individual P450s often present characteristic cell type- and tissue-specific expression patterns. As an extrahepatic form of cytochrome P450, CYP4B1 is predominantly expressed in the lung, as well as in other organs in smaller amounts [8]. The contribution of CYP4B1 in cancer is of particular interest, as its expression was found to be altered in a couple of specific cancers, including lung cancer [9–14]. As the primary site of exposure to inhaled toxicants and carcinogens, the metabolic balance of the protective detoxification system of the lung organ is essential to maintain its normal physiological function; thus, it is reasonable to speculate that the dysregulation of CYP4B1 is presumably associated with carcinogenesis in the lung owing to its catalytic activity in the first step of xenobiotic processing. Accordingly, in the present study, using data from The Cancer Genome Atlas (TCGA) project and the Gene Expression Omnibus (GEO) database, we performed a secondary analysis to thoroughly analyze the *CYP4B1* expression level, determine its prognostic role, explore the underlying mechanisms of its dysregulation, as well as to probe its potential functions in LUAD.

## 2. Materials and methods

### 2.1. Data mining

In this retrospective study, gene expression data with clinical information from the TCGA-LUAD project were obtained for this retrospective study from its official website (https://www.cancer.gov/about-nci/organization/ccg/research/structural-genomics/tcga). The exclusion criteria were patients who did not present primary tumors and/or had received neoadjuvant therapy. Tissues from 511 patients with primary tumors and 59 adjacent normal counterparts were extracted for RNA sequencing using Illumina HiSeq. To measure the amount of gene expression, level 3 normalized sequencing data of fragments per kilobase of transcript per million mapped reads (FPKM) were used. Of the 511 patients, 501 had intact overall survival (OS) data recorded, while 438 had intact recurrence-free survival (RFS) data recorded. Clinicopathological parameters including age, sex, pathological stage, radiation therapy, pharmaceutical therapy, tobacco smoking history, and canonical mutations in *KRAS/EGFR/ALK* were collected. The age of the patients at diagnosis ranged from 33 to 88 years. The pathological stage, reflecting the extent of the cancer, was graded as I, II, III, and IV based on the American Joint Committee on Cancer staging criteria [15]. The tobacco smoking history was categorized as level 1–5, describing the smoking status and history as self-reported by a patient, with level 1 indicating lifelong non-smokers, while levels 2–5 representing current smokers with different lengths of smoking. Additionally, living status, OS in days, recurrence status, and RFS in days were also downloaded for survival-related analysis. Three gene microarray datasets of lung cancer (GSE30219, GSE31210 and GSE32863) were retrieved from the GEO database (http://www.ncbi.nlm.nih.gov/geo/) for validation. To explore the underlying mechanisms of *CYP4B1* dysregulation in LUAD, somatic mutations, linear copy number alterations (CNAs), and DNA methylation status in *CYP4B1* were simultaneously collected from TCGA.

### 2.2. Functional and pathway enrichment analysis

The LinkedOmics database is a unique platform containing multi-omics data and clinical data for 32 cancer types from the TCGA project, allowing biologists and clinicians to access, analyze and compare cancer multi-omics data within and across tumor types [16]. To investigate the underlying function of *CYP4B1* in LUAD, we identified genes differentially co-expressed with *CYP4B1* expression in TCGA-LUAD via LinkedOmics. The cut-off criteria of studies were set as a Benjamini and Hochberg false discovery rate (FDR) < 0.05 and a |Pearson’s r| > 0.2. GEPIA2 (http://gepia2.cancer-pku.cn) web server is a valuable and highly cited resource for gene expression analysis based on tumor and normal samples from the TCGA and GTEx databases [17]. In our study, it was used for further analysis of the top three genes positively correlated with *CYP4B1* identified by LinkedOmics, involving their expression profiles and prognostic value in LUAD. Gene ontology (GO) analysis and gene set enrichment analysis (GSEA), categorized by the Kyoto Encyclopedia of Genes and Genomes (KEGG) pathway was performed using the LinkInterpreter module of LinkedOmics. FDR < 0.05 indicates statistically significant.

### 2.3. Statistical analysis

SPSS Statistics 20 (SPSS Inc., IL, USA) and GraphPad Prism 8 (GraphPad Inc., CA, USA) were used for statistical analyses. Comparison between the two groups and the association of *CYP4B1* expression with clinicopathological parameters was evaluated using Welch’s *t*-test. Gene expression levels were categorized as low or high according to median values. Receiver operating characteristic (ROC) analysis was performed to assess the diagnostic value of *CYP4B1* expression in LUAD. Kaplan-Meier curves of OS and RFS were generated using GraphPad Prism by setting the median *CYP4B1* expression as the cut-off. A log-rank test was performed to examine the significance of differences between curves. The prognostic value was analyzed using univariate and multivariate Cox regression models. Factors correlated with OS outcomes in the univariate analysis were included in the multivariate analysis. Pearson correlation coefficients were calculated to evaluate the correlations between the two groups of continuous variables. *p*<0.05 indicates statistical significance for all statistical analyses performed.

## 3. Results

### 3.1. *CPY4B1* was significantly downregulated in patients with LUAD

**Fig 1:**
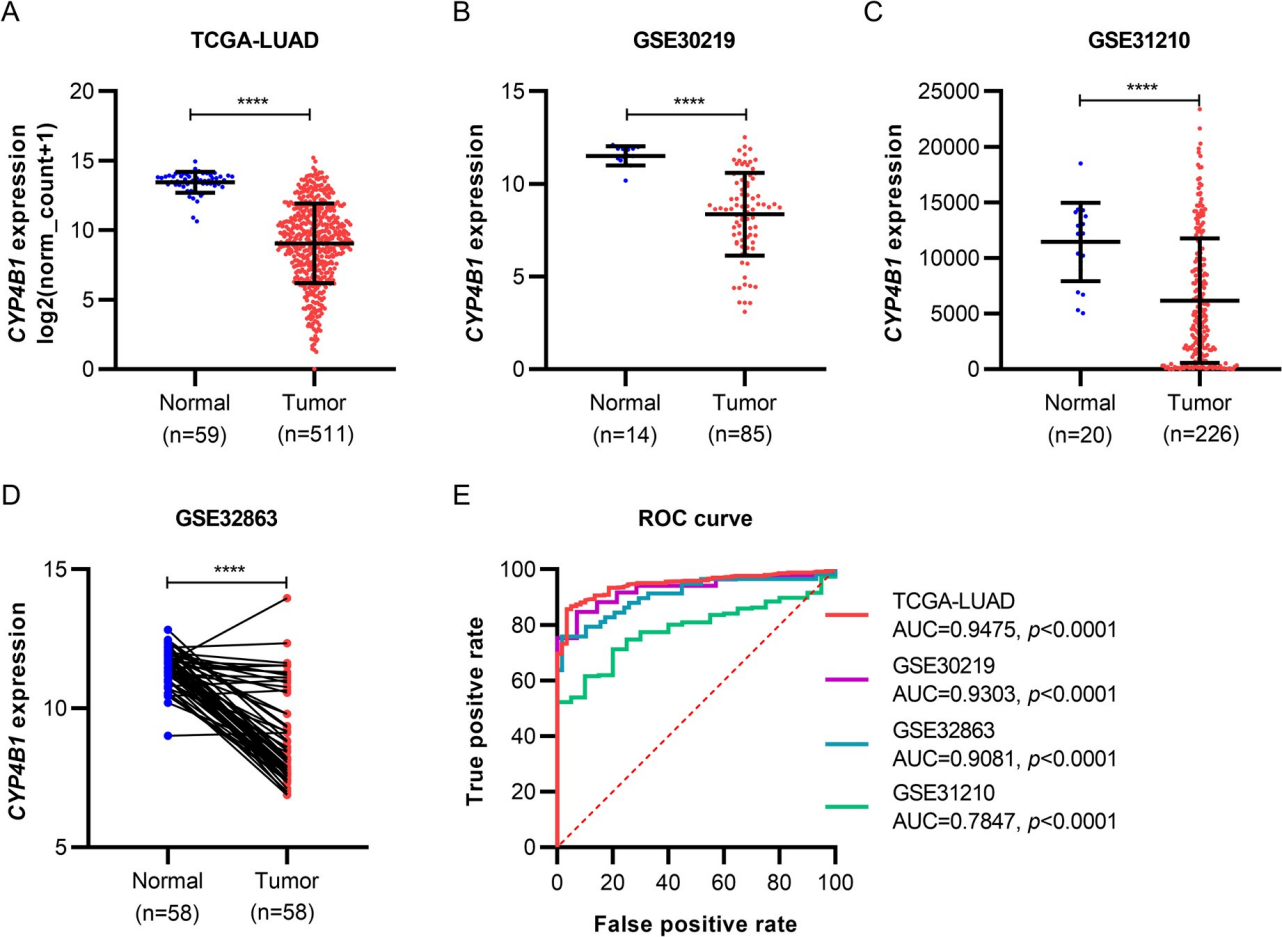
*CYP4B1* mRNA expression profiles in patients with LUAD from TCGA and GEO dataset cohorts. (A–D) *CYP4B1* mRNA expression in LUAD tissues and normal controls from a TCGA cohort (A), GSE30219 cohort (B), GSE31210 cohort (C) and GSE32863 cohort (D). (E) ROC curves showing the diagnostic value of *CYP4B1* in LUAD (red curve, data from TCGA cohort; purple curve, data from GSE30219 cohort; blue curve, data from GSE32863 cohort; green curve, data from GSE31210 cohort). ****p<0.0001. LUAD, lung adenocarcinoma; TCGA, The Cancer Genome Atlas; GEO, Gene Expression Omnibus; ROC, receiver operating characteristic.

To examine the mRNA expression level of *CYP4B1* in patients with LUAD, we extracted RNA-seq data from the TCGA project and microarray data of GSE30219, GSE31210, and GSE32863 from the GEO database for validation. In the TCGA-LUAD cohort, the results indicated that *CYP4B1* mRNA expression was approximately 0.33-fold lower expressed in tumor tissues (n = 511) than in adjacent normal tissues (n = 59) (Fig 1A). Next, this finding was verified in specimens from the three GEO datasets (Fig 1B–1D). Moreover, the area under the curve (AUC) value of *CYP4B1* expression for LUAD diagnosis was no less than 0.7847 in the four LUAD cohorts, all with a *p*-value<0.0001 (Fig 1E).

Next, we explored the expression pattern of *CYP4B1* in different subgroups of clinicopathological parameters. The results revealed that the significantly decreased *CYP4B1* expression was more frequently observed in patients with age younger than 65 years (*p* = 0.0041), in those with a history of pharmaceutical therapy (*p* = 0.0127) and radiation therapy (*p* = 0.0340), mutations in *KRAS/EGFR/ALK* (*p* = 0.0239), and deaths (i.e., living status; *p* = 0.0026) (Table 1).

**Table 1:** Correlation between the clinicopathological parameters and *CYP4B1* expression in LUAD.

Parameters	Variables	n	Mean ± SD	t	*p-*value
Age (years)	<65	220	8.588±2.896	2.886	0.0041
	≥65	275	9.339±2.854		
Gender	Female	277	9.255±2.778	1.793	0.0736
	Male	237	8.801±2.961		
Smoking history	1	75	9.607±3.153	1.804	0.0719
	2/3/4/5	425	8.963±2.793		
Pathological stage	I/II	396	9.162±2.863	1.716	0.0867
	III/IV	110	8.634±2.817		
Pharmaceutical therapy	No	280	9.304±2.994	2.504	0.0127
	Yes	188	8.653±2.586		
Radiation therapy	No	362	9.150±2.876	2.127	0.0340
	Yes	107	8.308±2.762		
Mutations in *KRAS/EGFR/ALK*	No	134	8.938±2.842	2.274	0.0239
	Yes	96	9.756±2.572		
Recurrence status	No	275	9.103±2.953	0.932	0.3523
	Yes	162	8.838±2.826		
Living status	Living	327	9.340±2.767	3.036	0.0026
	Dead	187	8.531±2.980		

LUAD, lung adenocarcinoma; SD, standard deviation.

### 3.2. Low *CYP4B1* expression was associated with poor outcomes of patients with LUAD

**Fig 2:**
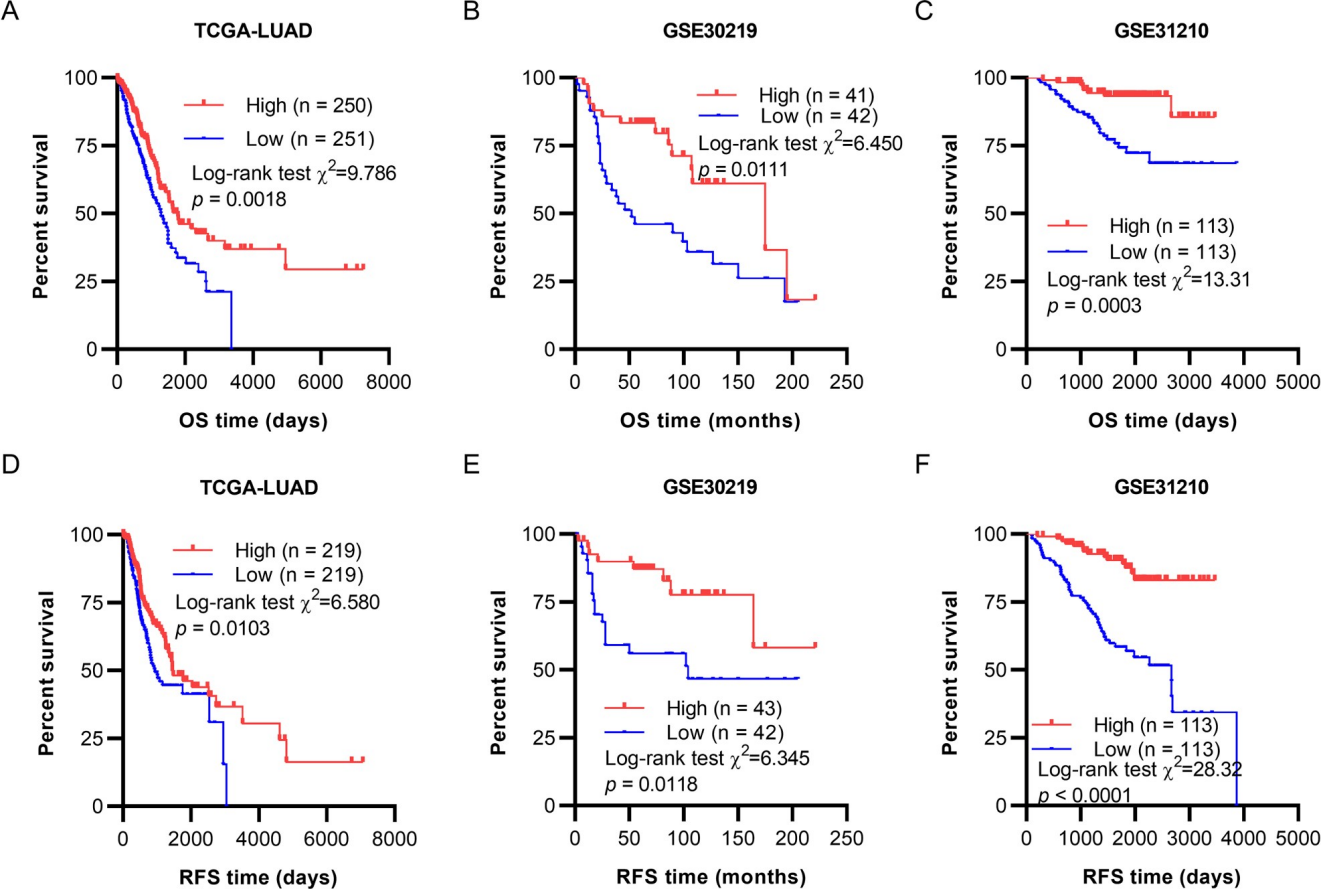
Low *CYP4B1* expression was associated with unfavorable survival in patients with LUAD. (A–C) Kaplan-Meier curves of OS in patients with LUAD from a TCGA cohort (A), GSE30219 cohort (B) and GSE31210 cohort (C). (D-F) Kaplan-Meier curves of RFS in patients with LUAD from a TCGA cohort (D), GSE30219 cohort (E) and GSE31210 cohort (F). LUAD, lung adenocarcinoma; OS, overall survival; TCGA, The Cancer Genome Atlas; RFS, recurrence-free survival.

To assess the correlations between *CYP4B1* mRNA expression and survival outcomes in LUAD patients, Kaplan-Meier curves were generated using clinical survival data of OS and RFS from TCGA. As shown in Fig 2A and 2D, low *CYP4B1* expression was more strongly associated with shorter OS and RFS in patients with primary LUAD (*p* = 0.0018 and *p* = 0.0103, respectively). On using GEO datasets for validation, the decreased *CYP4B1* expression group was found to possess remarkably inferior OS and RFS when compared with the high *CYP4B1* expression group in both GSE30219 (*p* = 0.0111 and *p* = 0.0118, respectively; Fig 2B and 2E) and GSE31210 (*p* = 0.0003 and *p*<0.0001, respectively; Fig 2C & 2F) datasets.

**Fig 3:**
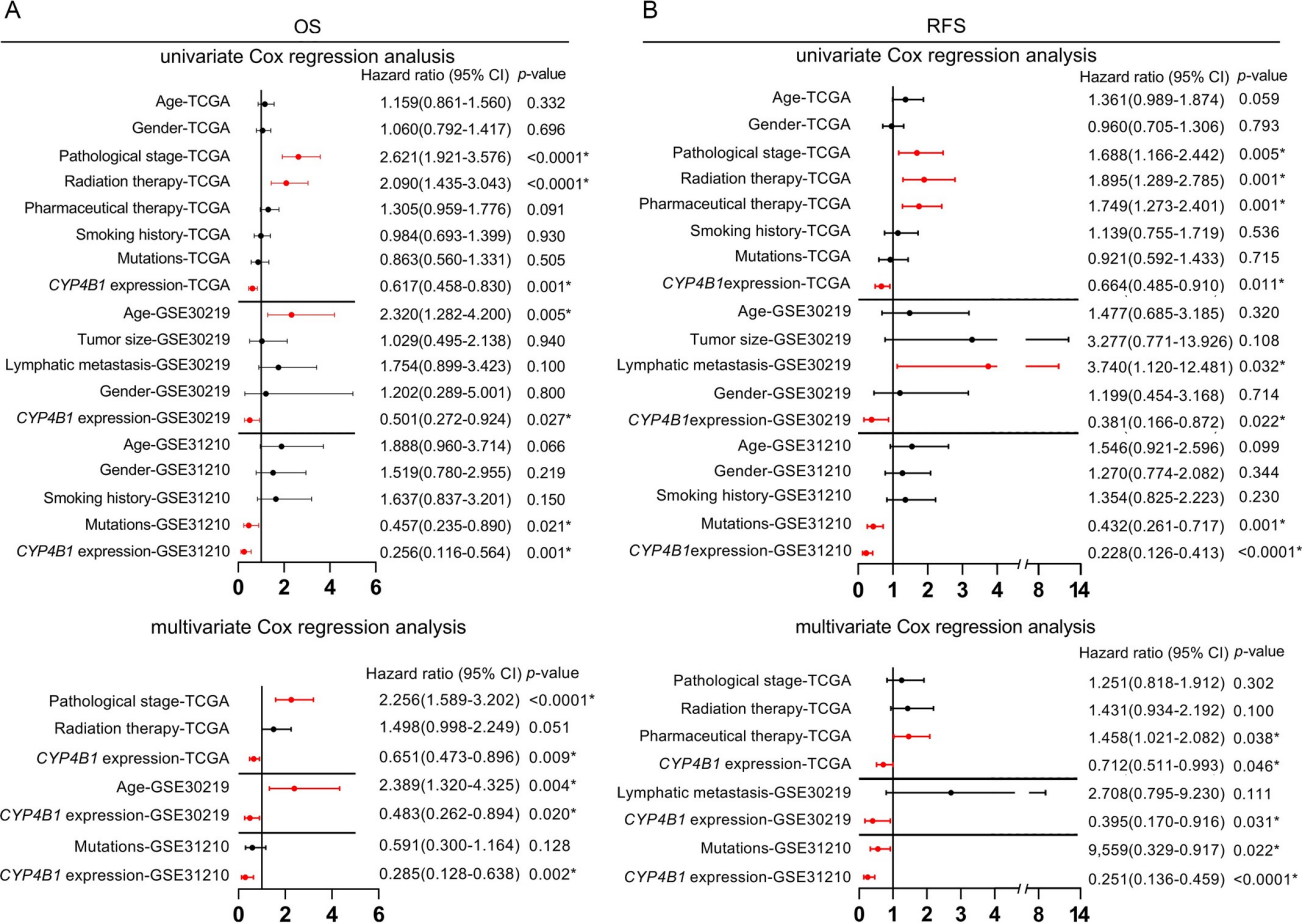
Forest plots of Cox regression analysis. (A) Forest plots showing univariate and multivariate analysis of OS in patients with LUAD. (B) Forest plots showing univariate and multivariate analysis of RFS in patients with LUAD. *p<0.05. OS, overall survival; LUAD, lung adenocarcinoma; RFS, recurrence-free survival.

Moreover, to verify the robust prognostic value of *CYP4B1* in terms of OS and RFS, univariate and multivariate analyses based on the Cox regression model were performed. Regarding OS, the decreased *CYP4B1* expression was found to be linked with unfavorable OS in patients from the TCGA-LUAD cohort via univariate analysis (*p* = 0.001, Fig 3A). Multivariate analysis confirmed that low *CYP4B1* expression could independently predict poor OS after adjustment for risk factors correlated with OS outcomes in univariate analysis (*p* = 0.009, Fig 3A). Subsequently, the same analysis performed on datasets from the GEO database further verified that low *CYP4B1* expression was an independent prognostic indicator (*p*_GSE30219_ = 0.020 and *p*_GSE31210_ = 0.002, respectively; Fig 3A). In terms of RFS, the independent prognostic value of *CYP4B1* in patients with LUAD was observed in the TCGA-LUAD cohort (*p* = 0.046) and was validated in the two independent GEO datasets (*p*_GSE30219_ = 0.031 and *p*_GSE31210_<0.0001, respectively; Fig 3B).

### 3.3. *CYP4B1* expression was regulated by CNAs and DNA methylation

**Fig 4:**
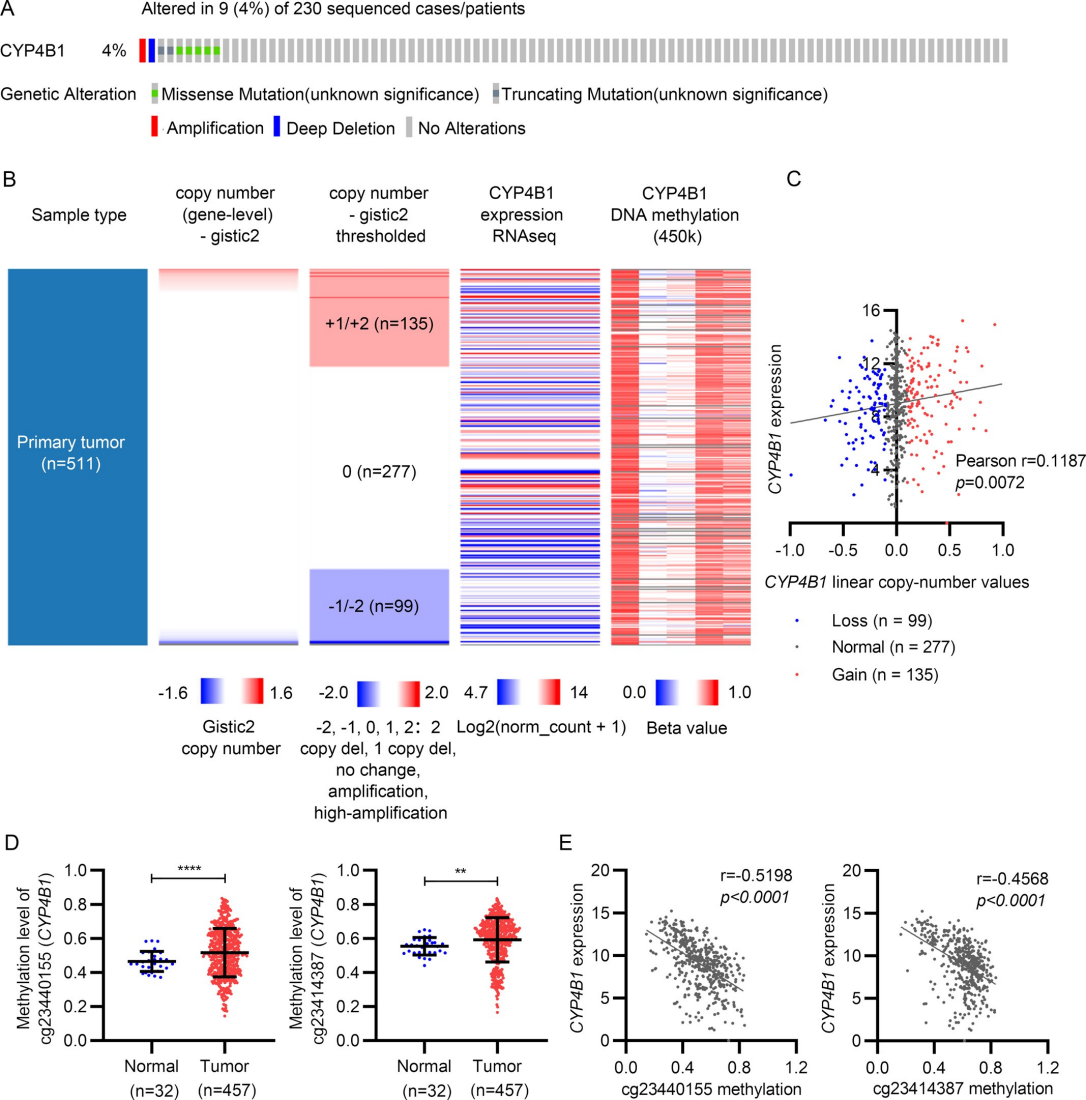
*CYP4B1* expression was modulated by its gene alterations and DNA methylation in LUAD. (A) Genetic alterations of *CYP4B1* in LUAD. (B) Heatmap of *CYP4B1* expression, DNA copy number alterations (gistic2 linear and gistic2 thresholded), and DNA methylation (Methylation 450k). (C) *CYP4B1* expression correlates with its linear copy number values by linear regression analysis. (D) DNA methylation level of cg23440155 and cg23414387 sites is significantly upregulated in LUAD tissues when compared with adjacent normal tissues. (E) *CYP4B1* expression correlates with cg23440155 and cg23414387 methylation levels by a linear regression analysis. LUAD, lung adenocarcinoma.

Next, we explored the potential mechanism of *CYP4B1* dysregulation. First, genetic alterations in *CYP4B1* were detected in TCGA-LUAD using the cBio-Portal for the Cancer Genomics platform (http://www.cbioportal.org/). *CYP4B1* mutations were observed in 9 out of 230 (4%) LUAD cases, with missense mutations as the main alteration type detected (Fig 4A). On analyzing *CYP4B1* DNA CNAs in 511 cases of LUAD, we observed that copy amplification occurred in 135 cases (+1/+2, 26.4%), copy deletion in 99 cases (-1/-2, 19.4%), and copy neutrality in 277 cases (0, 54.2%) (Fig 4B). Regression analysis demonstrated a significant correlation between *CYP4B1* expression and linear copy number values (Pearson’s r = 0.1187, *p* = 0.0072, Fig 4C). Additionally, the methylation status of five CpG sites in the *CYP4B1* gene was extracted to assess its role in regulating *CYP4B1* expression. By comparing the methylation level between tumor (n = 457) and normal tissues (n = 32), we observed that two CpG sites, cg23440155 and cg23414387, were remarkably hypermethylated in the tumor group (Fig 4D and S1 Table). In Pearson’s regression analysis, *CYP4B1* expression was found to be strongly and negatively correlated with DNA methylation in the two selected CpG sites (cg23440155: Pearson’s r = -0.5198, *p*<0.0001; cg23414387: Pearson’s r = -0.4568, *p*<0.0001) (Fig 4E).

### 3.4. Gene set enrichment analysis of genes co-expressed with *CYP4B1* in LUAD

**Fig 5:**
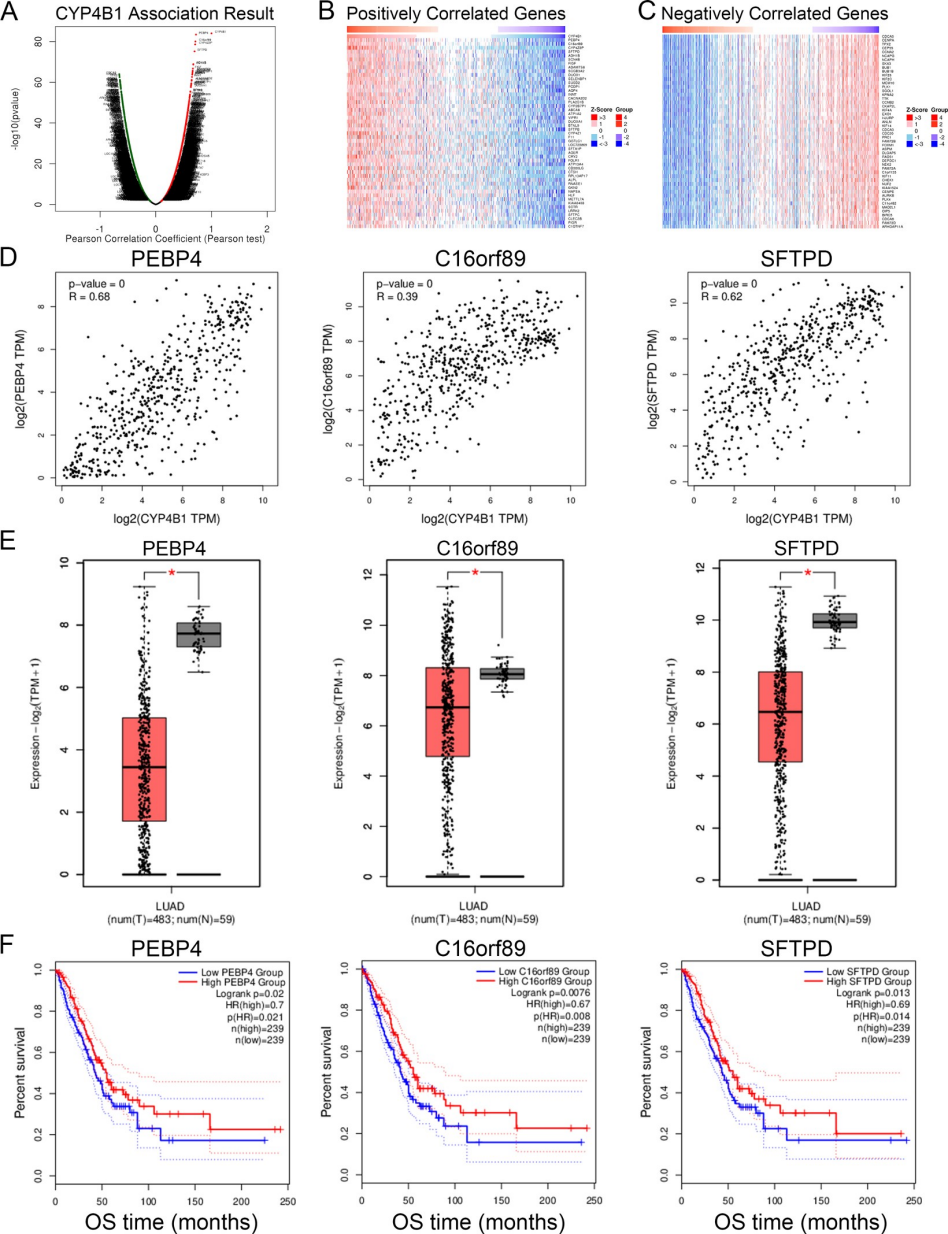
Differentially expressed genes correlated with *CYP4B1* in LUAD. (A) Volcano plot showing differentially expressed genes significantly associated with *CYP4B1* (FDR<0.05; |Pearson’s r|>0.2). (B, C) Heatmaps showing the top 50 significant genes positively and negatively correlated with *CYP4B1* in LUAD. (D-F) The top three significant genes demonstrating positive co-expression with *CYP4B1* in LUAD. (D) Pearson correlation of *CYP4B1* expression with *PEBP4*, *C16orf89*, and *SFTPD*. (E) The mRNA expression profiles of *PEBP4*, *C16orf89*, and *SFTPD* in LUAD tissues when compared with normal lung tissues. (F) Survival analysis of *PEBP4*, *C16orf89*, and *SFTPD* in LUAD. FDR, false discovery rate; LUAD, lung adenocarcinoma.

By data mining using the LinkFinder module of LinkedOmics, we identified the genes co-expressed with *CYP4B1* in 515 patients with LUAD from the TCGA project (FDR<0.05, and (|Pearson’s r|)>0.2). The results indicated that a lot of 3829 and 2516 genes were positively and negatively correlated with *CYP4B1* expression, respectively (Fig 5A). A heatmap of the top 50 significant genes positively or negatively associated with *CYP4B1* is shown in Fig 5B and 5C. Among them, expression of *PEBP4*, *C16orf89* and *SFTPD* showed the strongest positive correlation with *CYP4B1* expression (*PEBP4*: Pearson’s r = 0.68; *C16orf89*: Pearson’s r = 0.39; *SFTPD*: Pearson’s r = 0.62) (Fig 5D). Consistent with *CYP4B1* expression, the three genes mentioned above revealed lower expression in tumor tissues than in their normal counterparts. Meanwhile, the decreased expression groups were linked to an inferior OS in patients with LUAD (Fig 5E and 5F).

**Fig 6:**
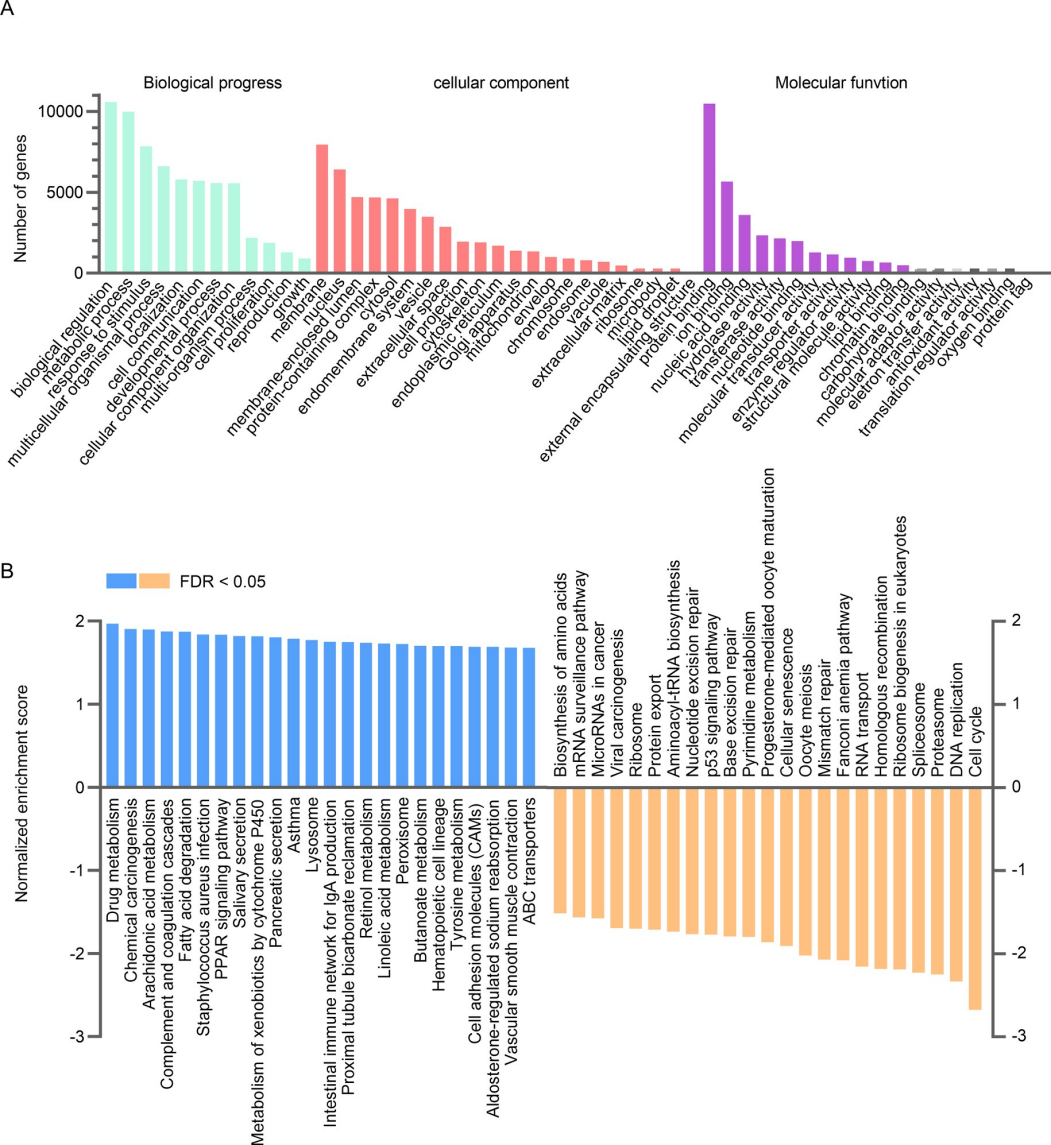
Gene ontology and GSEA analysis categorized by the KEGG pathway of the genes co-expressed with *CYP4B1* in TCGA-LUAD. GSEA, Gene set enrichment analysis; KEGG, Kyoto Encyclopedia of Genes and Genomes; TCGA, The Cancer Genome Atlas; LUAD, lung adenocarcinoma.

To further investigate the possible gene ontology terms and signaling pathways in which *CYP4B1* might be involved, GO and GSEA analyses were performed using the LinkInterpreter module of LinkedOmics. The results of GO analysis showed that genes positively co-expressed with *CYP4B1* were mainly located in the membrane, nucleus and membrane-enclosed lumen, and generally participated in biological processes of biological regulation, metabolic processes and responses to stimuli by molecular functions such as protein binding, ion binding, and nucleic acid binding (Fig 6A). Furthermore, the GSEA analysis revealed that the top five enriched KEGG pathways positively linked with *CYP4B1* expression were drug metabolism, chemical carcinogenesis, arachidonic acid metabolism, complement and coagulation cascades and fatty acid degradation, while the most negatively correlated pathway was the cell cycle (Fig 6B).

## 4. Discussion

CYP4B1, a member of the CYP450s superfamily, is responsible for the oxidative metabolism of a series of both endogenous compounds and xenobiotics, preferentially via a ω-hydroxylation pathway [18]. The gene sequence of CYP4B1 has been evolutionarily conserved, and the enzyme has been detected by immunoblotting in animals such as rabbits, guinea, mice, monkeys, and hamsters [18, 19]. The expression of CYP4B1 is predominantly extrahepatic, presenting tissue and species specificity. Studies have revealed that the human forms of CYP4B1 possess limited activity *in vivo* when compared with animals, rendering rabbit CYP4B1 a feasible gene therapy in humans to treat cancers in humans [20]. To date, the physiological function of innate human CYP4B1 remains unclear. Nevertheless, like other CYP4 proteins, CYP4B1 can metabolize endogenous fatty acids and hydrocarbons, as well as participates in the bioactivation of a wide variety of xenobiotics, including valproic acid (VPA), 2-aminofluorene (2-AF), 3-methylindole (3-MI), 4-ipomeanol (4-IPO) and numerous aromatic amines in rodents and ruminants. The CYP4B1 catalytic metabolites or reactive intermediates of these xenobiotics reportedly exert toxicological effects by forming adducts with DNA or proteins, eliciting organ-specific toxicities, which might contribute to the formation and progression of carcinogenesis. One example is that a high expression of CYP4B1 increases the risk of bladder tumor by activation of 2-AF [10].

Czerwinski et al. have first quantified *CYP4B1* mRNA in the human lung, as well as in lung tumors, and observed 2.3-fold lower mRNA levels in tumors [9]. However, to our knowledge, the expression profile and related function of *CYP4B1* have not been studied in LUAD species. In this study, we confirmed the carcinogenic role of *CYP4B1* in LUAD for the first time. By analyzing RNA-seq and microarray data of LUAD, *CYP4B1* was found to demonstrate a remarkably lower expression in tumor specimens than in noncancerous lung tissues. Meanwhile, the AUC value for *CYP4B1* was no less than 0.7847 in the four LUAD cohorts of TCGA-LUAD, GSE30219, GSE32863, and GSE31210, revealing that the gene expression signature of *CYP4B1* is a useful marker for LUAD diagnosis.

Several studies have explored the role and significance of dysregulated CYP4B1 in human cancers. In adrenocortical carcinoma (ACC), *CYP4B1* expression was nearly absent when compared with that in the normal adrenal cortex. Ectopic expression of *CYP4B1* reportedly promotes cytotoxicity and increases chemosensitivity in ACC cell lines, implicating the role of *CYP4B1* in tumorigenesis and chemoresistance in ACC [12]. Upon injury to the cornea, the *CYP4B1* enzymatic pathway was found to be activated, leading to the production of 12-hydroxyeicosatrienoic acid (12-HETrE), a potent inflammatory and angiogenic eicosanoid. By applying siRNA duplexes targeting *CYP4B1 in vitro*, the neovascular response and expression of corneal *VEGF* were notably reduced. These results strongly suggest that corneal *CYP4B1* is a component of the inflammatory and neovascular cascade initiated by injury to the corneal epithelium [14]. Moreover, downregulation of CYP4B1 protein levels in urothelial carcinomas (UC) was found to be correlated with several clinicopathological factors of an advanced primary tumor, nodal metastasis, high histological grade, vascular invasion, perineural invasion, and mitotic rate. Loss of CYP4B1 expression is an independent unfavorable prognosticator of UC [13].

Currently, the relevance of the association between *CYP4B1* expression and the clinical features of LUAD remains unclear. Our findings revealed that low *CYP4B1* mRNA expression was linked to factors such as age less than 65 years, history of pharmaceutical therapy and radiation therapy, mutations in *KRAS/EGFR/ALK*, and death (living status). The correlation between *CYP4B1* expression and a history of pharmaceutical therapy has implicated its crucial role in drug metabolism in LUAD. Notably, Murtha et al. have observed that the exogenous expression of *CYP4B1* accentuates the apparent cytotoxicity in ACC cell lines that were treated with mitotane or cisplatin [12]. As LUAD is a drug-resistant tumor, this finding could present *CYP4B1* as an attractive future target for resistant-LUAD therapeutics. Kaplan-Meier survival analyses indicated that patients with low *CYP4B1* expression had remarkably shorter OS and RFS. By performing univariate and multivariate analyses, we confirmed that decreased *CYP4B1* RNA expression was independently associated with inferior OS and RFS in patients with LUAD. Therefore, we infer that reduced *CYP4B1* expression could serve as an independent predictor of unfavorable OS and RFS.

It is well recognized that the drug-mediated activation of *CYP* gene is regulated by various members of the nuclear receptor superfamily. For *CYP4B1*, computational analysis of its promoter region from rabbit cornea revealed sequences that could potentially bind heterodimers of the retinoic acid receptors (RARs), including retinoic acid receptor/retinoid X receptor (RAR/RXR), vitamin D receptor/RXR (VDR/RXR), and peroxisome proliferator-activated receptor/retinoid X receptor (PPAR/RXR). We then verified that the transcription of *CYP4B1* in HepG2 cells was significantly activated by incubation with retinoic acid [21]. Furthermore, studies have observed that the binding activity of the promoter region of rabbit corneal *CYP4B1* was induced under hypoxic conditions for hypoxia-inducible factor-1 (HIF-1), nuclear factor-kappa light chain enhancer of activated B cells (NF-κB), and activator protein-1 (AP-1) [22]. In addition to regulation at the transcriptional level, genetic and epigenetic alterations affect gene expression during carcinogenesis. In the present study, we observed that *CYP4B1* DNA alterations occurred in LUAD, in which missense mutations were the major type. Genetic variants of *CYP4B1* have been reported in several previous studies [23–26]. Among them, CYP4B1*2 was found to lead to a complete loss of *CYP4B1* function in a cohort of French Caucasians [23]. In a Japanese population, subjects carrying the CYP4B1*1/*2 or CYP4B1*2/*2 genotypes exhibited a 1.75-fold increased risk of bladder cancer [26]. These findings suggest that genetic alteration of *CYP4B1* could impact its own functionality. However, the DNA mutations observed based on TCGA-LUAD in our study are yet to be reported (data not shown). Thus, further studies are needed to evaluate inter-individual variations in the activity of CYP4B1 coding variants. Moreover, *CYP4B1* dysregulation was found to be regulated by copy number variation, as well as the hypermethylation of cg23440155 and cg23414387 sites.

Additionally, the prognostic role of *CYP4B1* indicated that it might act as a tumor-driven gene in LUAD. We then identified its co-expressed genes and performed the GO and GSEA enrichment analyses to explore the underlying oncogenic properties of *CYP4B1* in LUAD. In our study, three genes, *PEBP4*, *C16orf89*, and *SFTPD*, were found to be significantly and positively co-expressed with *CYP4B1*. These genes all showed remarkable downregulation in tumor tissues and were linked to the unfavorable OS in LUAD. *PEBP4* and *SFTPD* have been reported as oncogenes in lung cancer [27–33]. However, *C16orf89* is a rarely investigated gene and its role in LUAD needs to be further confirmed. As suggested by GO and GSEA enrichment analyses, genes positively co-expressed with *CYP4B1* were predominantly enriched in biological metabolism-related activities and regulation of exogenous and endogenous compounds, including drug metabolism, chemical carcinogenesis, and arachidonic acid metabolism. This finding is consistent with the physiological functions of *CYP4B1*. Moreover, one of the top pathways that was negatively correlated with *CYP4B1* expression was the cell cycle. Disruption of multiple biological pathways is a hallmark of several tumors, including LUAD. These results suggest that inhibition of *CYP4B1* in LUAD promotes tumorigenesis by preventing metabolism and enhancing DNA replication and cell cycle activities. Overall, our findings provide new insights into the role of *CYP4B1* in the development and treatment of LUAD. However, this study had several limitations. First, the number of subjects included in our study cohort was relatively small, which probably limits the statistical power, especially in the subgroup analyses. Second, we observed that both CNAs and DNA methylation contributed to the decreased expression of *CYP4B1* in LUAD; however, we were unable to determine whether additional genetic or epigenetic mechanisms influence its transcription. Third, the results of this study were not externally validated; thus, experimental studies are required to further explore the role and underlying mechanisms if action of *CYP4B1* in LUAD.

## 5. Conclusion

*CYP4B1* expression may serve as a valuable independent prognostic biomarker and a potential therapeutic target in patients with LUAD.

## Supporting information

S1 Table Methylation status of CpG sites in *CYP4B1* gene in LUAD patients. (DOCX)
