# Supplementary material for: CYP4B1 is a prognostic biomarker and potential therapeutic target in lung adenocarcinoma
Source: PLoS One. 2021 Feb 16;16(2):e0247020. doi: 10.1371/journal.pone.0247020 (PMC7886130; doi:10.1371/journal.pone.0247020)
Supplement: S1 Table — (DOCX) [file pone.0247020.s001.docx]

S1 Table. Methylation status of CpG sites in *CYP4B1* gene in LUAD patients.

| CpGs | Group | Mean (%) | ΔMean (%) | *p* value |
| --- | --- | --- | --- | --- |
| cg14222679 | Normal | 86.87 | 1.97 | 0.0040 |
|  | Tumor | 84.90 |  |  |
| cg23440155 | Normal | 46.50 | -5.23 | <0.0001 |
|  | Tumor | 51.73 |  |  |
| cg23414387 | Normal | 55.51 | -3.71 | 0.0012 |
|  | Tumor | 59.22 |  |  |
| cg15027165 | Normal | 85.20 | 5.64 | <0.0001 |
|  | Tumor | 79.56 |  |  |
| cg19092343 | Normal | 82.06 | 6.65 | <0.0001 |
|  | Tumor | 75.41 |  |  |

LUAD, lung adenocarcinoma.
